# N-linked glycosylation plays an essential role in the stability and function of tissue-nonspecific alkaline phosphatase

**DOI:** 10.1016/j.jbc.2025.111092

**Published:** 2025-12-20

**Authors:** Diana Atanasova, Ali Saad Kusay, Lavanya Moparthi, Stefan Koch, Mathias Haarhaus, Sonoko Narisawa, José Luis Millán, Eva Landberg, Per Magnusson

**Affiliations:** 1 Department of Clinical Chemistry, and Department of Biomedical and Clinical Sciences, Linköping University, Linköping, Sweden; 2 Department of Biomedical and Clinical Sciences, Linköping University, Linköping, Sweden; 3 Wallenberg Centre for Molecular Medicine, Linköping University, Linköping, Sweden; 4 Division of Renal Medicine, Department of Clinical Science, Intervention and Technology, Karolinska Institutet, Karolinska University Hospital, Stockholm, Sweden; 5 Diaverum Sweden AB, Malmö, Sweden; 6 Sanford Children’s Health Research Center, Sanford Burnham Prebys Medical Discovery Institute, La Jolla, California, USA

**Keywords:** biomarker, biomineralization, cell-surface enzyme, computer modeling, enzyme kinetics, glycoprotein secretion, molecular dynamics, mutagenesis *in vitro*, N-linked glycosylation, protein stability

## Abstract

Tissue-nonspecific alkaline phosphatase (TNALP) is a membrane-anchored glycoprotein with five N-linked glycosylation sites (N140, N230, N271, N303, N430) that is crucial for bone mineralization. TNALP is released into the bloodstream, serving as a biomarker for bone and mineral disorders. This study explores the role of N-linked glycosylation in the secretion, enzymatic activity, stability, and structure of TNALP. To eliminate the N-linked glycosylation site specifically, a soluble TNALP expression construct was created with the following substitution mutations N140Q, N230Q, N271Q, N303Q and N430D, and expressed in mouse osteoblasts. The effect of glycosylation was also studied by computational modeling (molecular dynamics simulations and the GlycoSHIELD tool). We observed that substituting glycosylation sites reduced TNALP secretion, particularly in the double-site mutations N140Q/N271Q and N230Q/N271Q, due to increased cellular retention. Mutations comprising site N271 (N271Q, N140Q/N271Q, N271Q/N303Q and N271Q/N430D) significantly impaired the enzymatic activity. The computational modeling indicated that N-glycans can stabilize regions of the protein, including the Ca^2+^-binding domain. Further, interactions between N-glycans can compensate for specific double-site glycan losses. Protein thermal stability analysis showed that, compared to WT, N271Q/N430D and N303Q/N430D had increased stability at 56°C. TNALP isoform analysis revealed no differences in isoform patterns for mutations with retained enzymatic activity. The study suggests that N-linked glycosylation, particularly the presence of glycans at N271, is vital for TNALP stability, secretion, and enzymatic function, offering insights into the structural and functional properties of TNALP.

Tissue-nonspecific alkaline phosphatase (TNALP) is a membrane-bound homodimeric glycoprotein expressed in various tissues but mainly in bone, liver and kidney. TNALP expressed by osteoblasts inactivates the mineralization inhibitor inorganic pyrophosphate and hydrolyzes ATP to provide inorganic phosphate for mineral formation and thereby initiates biomineralization (1, 2, 3). Hydrolysis of the glycosylphosphatidylinositol (GPI) anchor by phospholipase D releases TNALP from cell membranes into the circulation, where the liver and bone isoforms under physiological conditions comprise the major part of the total alkaline phosphatase (ALP) activity in a 1:1 ratio (4). TNALP in the circulation is involved in lipid metabolism by dephosphorylating phosphocholine to provide choline for very low-density lipoprotein particle production by hepatocytes (5). Bone TNALP in blood can be used as a biomarker of bone formation. Liver and bone TNALP, considered isoforms (or glycoforms) of TNALP, differ in their post-translational N- and O-linked glycosylation (4, 6). Bone TNALP expressed in osteoblasts encompasses four isoforms based on HPLC with anion-exchange chromatography, though the difference in glycosylation pattern between these isoforms is still largely unknown (6). TNALP has five N-linked glycosylation sites located at N140, N230, N271, N303 and N430 (7). We have recently demonstrated that TNALP expressed by osteoblasts is fully glycosylated with various N-glycan structures on each site, predominantly biantennary complex-type N-glycans (8).

Lack of TNALP activity due to loss-of-function mutations in the *ALPL* gene causes the autosomal recessive inherited disease hypophosphatasia (HPP). More than 470 variants with various effects on TNALP function and skeletal phenotypes of varying degrees of severity have been reported (9, 10). In addition to low serum TNALP activity, elevated levels of the endogenous TNALP substrates pyrophosphate and pyridoxal-5′-phosphate are present in HPP patients. The severity of HPP varies from perinatal HPP and the most severe to milder forms of adult HPP and odontohypophosphatasia. Severe forms of perinatal and infantile HPP cause skeletal hypomineralization, epileptic seizures and premature death, while the symptoms of childhood HPP vary from premature loss of deciduous teeth, failure to thrive, muscle weakness and high fracture rate. Osteomalacia, impaired fracture healing and musculoskeletal pain are common manifestations in individuals with adult HPP (9).

Mutations in the canonical sequence NXS/T, required for N-linked glycosylation, with a negative effect on TNALP function have been reported at sites T141 (11, 12), R272 (12, 13), T273 (12), N430 (14) and V431 (15). These variants are described as pathogenic (T141N, R272C, R272H, R272L, N430S, V431A) or likely pathogenic (T273M, V431D). In the NXS/T sequon, X is any amino acid except proline, whereas serine or threonine is present at the third amino acid position. This sequon is essential for glycosyltransferases to form a glycosidic bond with the asparagine (N) in that sequence. It was found that individual amino acid substitution of the N-glycan sites did not significantly affect expression, dimer stability or enzymatic activity of membrane-bound TNALP, except N430Q. For this study, the N430D substitution was performed (instead of N430Q), since N430D has been shown to preserve dimer stability and enzymatic activity in membrane-bound TNALP (14). However, N230, N271 and N303 were collectively required for expression of functional TNALP, but it is not known whether the amino acid substitution or the lack of glycan at these sites is the cause of impaired protein stability (14). Previous studies have explored the molecular stability of TNALP by its ability to dimerize, tetramerize or octamerize in different soluble states (16, 17, 18). Glycosylation is important for protein folding and compartmentalization of membrane-bound and soluble proteins, such as TNALP (19, 20). Moreover, the effect of individual N-linked glycans on the stability, secretion and isoform profile of soluble TNALP remains unexplored. Therefore, the aim of this study was to investigate how the elimination of one or more N-linked glycosylation sites affects the enzymatic activity, protein stability and isoform profile of soluble TNALP. We combined computational modeling of glycosylated and aglycosylated TNALP and site-directed mutagenesis of one, two or three N-glycan sites to elucidate the influence of site-specific N-linked glycosylation in the structural and functional properties of soluble TNALP.

## Results

### N-glycan site mutations of TNALP led to decreased enzymatic activity and TNALP secretion

The five N-glycan sites of TNALP were substituted with glutamine (N>Q) or aspartic acid (N>D) to obtain the following mutants: N140Q, N230Q, N271Q, N303Q and N430D in a plasmid expressing flag-tagged TNALP. Plasmids with single- and double-site mutations were expressed in *Alpl*^*+/−*^ mouse osteoblasts. Homodimeric mutants were obtained by transfection with mutant-only plasmid, while heterodimeric mutants were obtained by transfection with mutant-to-WT plasmid in a 1:1 ratio.

**Figure 1:**
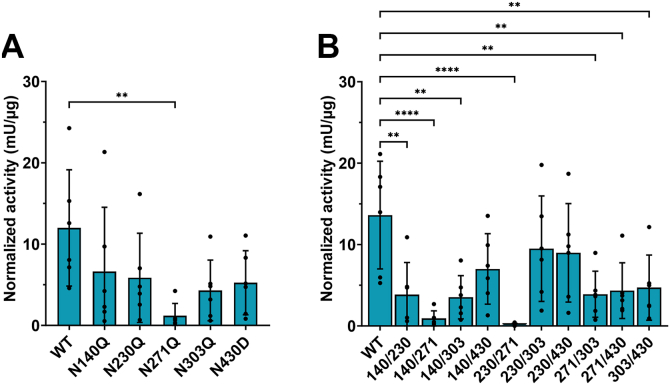
**Enzymatic activity of TNALP N-glycan site mutations.** (*A*) single-site and (*B*) double-site mutations. Cell supernatants were collected 2 days after transfection with WT and mutated TNALP-Flag in six independent transfection experiments. TNALP activity was measured with enzymatic activity assay with p-nitrophenylphosphate in diethanolamine buffer at pH 9.8. Bars represent mean ± SD, N = 6; one-way ANOVA with Dunnett’s multiple comparisons test, *p* < 0.05 considered as significant. Significant decrease in enzymatic activity was observed between WT and N271Q, and WT and most of the double-site mutations. ∗∗*p* < 0.007, ∗∗∗∗*p* < 0.0001. TNALP, tissue-nonspecific alkaline phosphatase.

**Figure 2:**
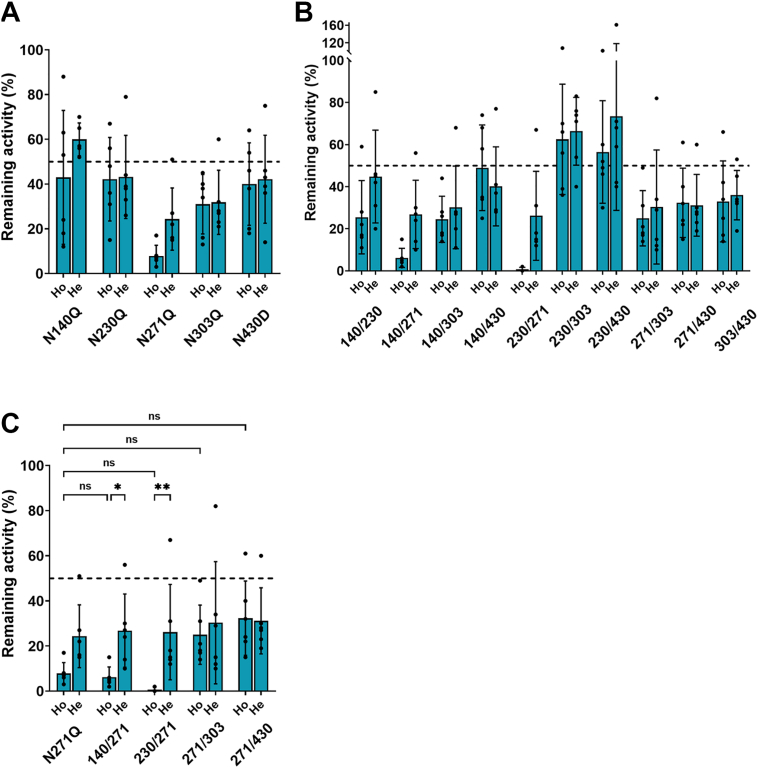
**Remaining TNALP activity after single- and double-site mutations of the N-glycan sites, relative to wild-type.** Both homodimeric (Ho) and heterodimeric (He) mutations were expressed after transfection with mutated-only or WT-to-mutated plasmid in 1:1 ratio, respectively, to determine dominant negative effect (<50% remaining activity). *A,* no significant differences in single-site Ho *versus*. He mutations. *B,* no significant differences between double-site Ho *versus*. He mutations, except in the N140Q/N271Q and N230Q/N271Q (also shown in (*C*)). *C,* no significant differences were seen in double-site mutations containing N271Q *versus* N271Q alone. Significant difference in remaining activity between the Ho *versus* He mutations was seen in N271Q in combination with N140Q (*p* = 0.02) or N230Q (*p* = 0.006). Bars represent mean ± SD of six independent replicate transfections; two-way ANOVA with Tukey’s multiple comparisons test, *p* < 0.05 considered as significant. ∗*p* = 0.04, ∗∗*p* = 0.007, ns: non-significant. Ho,homodimeric; He, heterodimeric; TNALP, tissue-nonspecific alkaline phosphatase.

The enzymatic activity in the single-site mutations in both monomers (homodimeric mutants) was significantly decreased in N271Q in comparison with WT TNALP (mean ± SD: 1.2 ± 1.5 *versus* 12 ± 7.1 mU/μg, N271Q *versus* WT, *p* = 0.007, Fig. 1*A*). The activity in all double-site homodimeric mutants compared to WT was significantly decreased, except in N230Q/N303Q and N230Q/N430D (mean ± SD: 9.5 ± 6.5 and 9 ± 6 *versus* 13.6 ± 6.6 mU/μg, Fig. 1*B*). The mean ± SD was 3.8 ± 3.9 mU/μg for N140Q/N230Q, 0.9 ± 0.9 mU/μg for N140Q/N271Q, 3.5 ± 2.7 mU/μg for N140Q/N303Q, 7 ± 4.3 mU/μg for N140Q/N430D, 0.15 ± 0.17 mU/μg for N230Q/N271Q, 3.9 ± 2.8 mU/μg for N271Q/N303Q, 4.3 ± 3.4 mU/μg for N271Q/N430D and 4.7 ± 4 mU/μg for N303Q/N430D *versus* 13.6 mU/μg ± 6.6 for WT, (*p* < 0.05, n = 6). To further evaluate whether the mutations have a dominant negative effect (DNE), *i.e.*, when a mutation in one monomer would influence the activity of the other monomer, heterodimeric mutants were generated. DNE is defined as less than 50% remaining activity relative to WT in the heterodimeric mutants. Figure 2 shows a comparison in the remaining activity between homodimeric and heterodimeric mutants. All single-site mutations showed drop in mean remaining activity under 50%, while N271Q and N303Q showed significant DNE with 24 ± 14% (CI: 10–39%) and 32 ± 14% (CI: 14–47%) (Fig. 2*A* and Table 1). All double-site homodimeric mutants, except N230Q/N303Q and N230Q/N430D, had a mean remaining activity under 50% and showed significant DNE, except N140Q/N430D (CI: 20–60%), N230Q/N303Q (CI: 49–83%) and N230Q/N430D (CI: 27–121%) (Fig. 2*B* and Table 1). Although the heterodimeric N140Q/N303Q (CI: 10–51%) and N271Q/N303Q did not show a significant DNE (CI: 2–59%), a high mean variance was observed, suggesting a tendency of DNE. All heterodimeric and homodimeric mutants of N271Q, with or without additional N-site substitution, led to the largest decrease in TNALP activity in comparison with all other mutants (Fig. 2*C*). Although, the homodimeric N271Q/N303Q and N271Q/N430D mutants had slightly enhanced activity in comparison with N271Q alone, there was no significant difference. There was a significant difference in the remaining activity of N140Q/N271Q (*p* = 0.04) and N230Q/N271Q (*p* = 0.007) between the homodimeric and heterodimeric mutants, showing a recovery in activity by the heterodimeric mutants. We also generated the triple-site mutations N140Q/N230Q/N430D, N230Q/N271Q/N303Q, and N230Q/N271Q/N430D but could not detect any activity.

**Table 1:** Dominant negative effect of heterodimeric mutations

Heterodimeric mutation	Remaining activity mean (±SD), %^a^	Confidence interval^b^ (5–95%)
N140Q	60 (7)	51–69
N230Q	43 (19)	24–63
N271Q	24 (14)	10–39
N303Q	32 (14)	17–47
N430D	42 (20)	22–63
N140Q/N230Q	45 (22)	22–68
N140Q/N271Q	27 (16)	10–44
N140Q/N303Q	30 (20)	10–51
N140Q/N430D	40 (19)	20–60
N230Q/N271Q	26 (21)	4–48
N230Q/N303Q	66 (16)	49–83
N230Q/N430D	74 (45)	27–121
N271Q/N303Q	30 (27)	2–59
N271Q/N430D	31 (15)	16–47
N303Q/N430D	36 (12)	24–48

a N = six.

b CI - values of 0 to 50% considered as significant for dominant negative effect.

**Figure 3:**
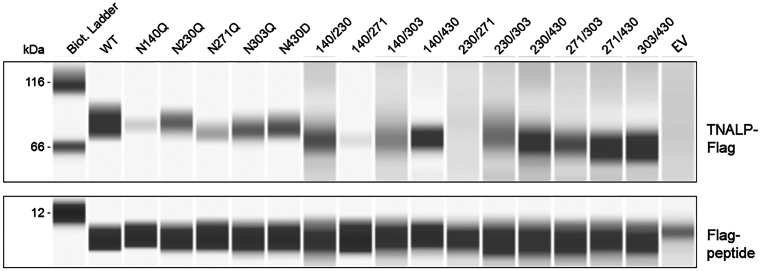
**Protein expression of soluble TNALP-Flag with single- and double-site homodimeric mutations.** Purified WT or mutated TNALP-Flag using Flag-immunoprecipitation with Flag-peptide elution from six replicate transfection experiments. The elutions were pooled together and immunoblotted with ProteinSimple immunoblotting assay (Bio-Techne) with rabbit polyclonal anti-Flag antibody. Residual Flag-peptide in the elutions was used as a loading control. EV: empty vector control. TNALP, tissue-nonspecific alkaline phosphatase.

**Figure 4:**
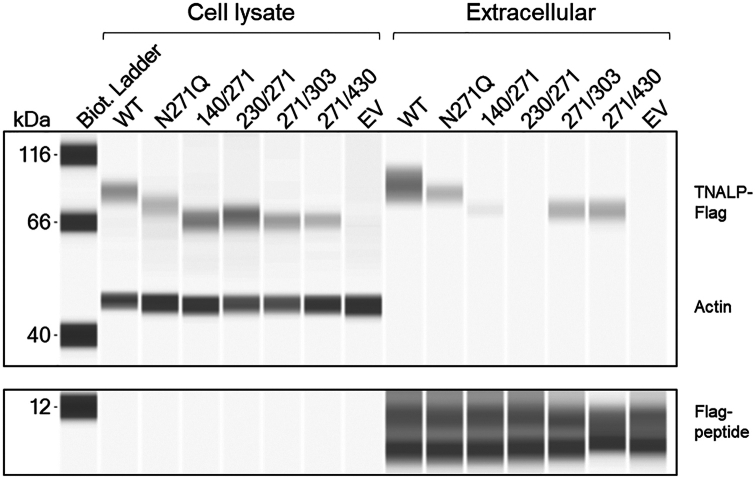
**Cellular retention of TNALP with N271Q single- and double-site homodimeric mutations.** Detection of TNALP-Flag in cell lysates and in cell supernatant (extracellular) of N271Q mutants 16 h post-transfection and culture with 1 μM MG-132. The cells were lysed with RIPA buffer and briefly sonicated. The extracellular TNALP-Flag was purified from cell medium using Flag-immunoprecipitation with Flag-peptide. Immunoblotting was performed with Protein Simple immunoblotting assay (Bio-Techne) with mouse monoclonal anti-Flag antibody. Residual Flag-peptide in the elutions was used as a loading control for extracellular TNALP-Flag. Actin was used as a loading control for cell lysates with mouse monoclonal anti-actin antibody. The image is representative of one independent experiment. EV, empty vector control; TNALP, tissue-nonspecific alkaline phosphatase.

To assess if N-glycan site mutations resulted in decreased extracellular release, TNALP was purified from cellular supernatants from six replicate transfections. The elutions were pooled, concentrated and immunoblotted (Fig. 3). All mutations led to slightly lower molecular weight in comparison with WT possibly due to loss of glycans (Table 2). Substitution of a single N-glycan site caused decreased protein levels in the supernatant relative to WT (fold-change <1, Table 2). No protein was detected in N230Q/N271Q (Fig. 3). To determine possible intracellular degradation of N230Q/N271Q and compare the variable amount of released protein, intracellular retention of TNALP was detected with immunoblotting in cell lysates. Initial analysis showed no presence of bands corresponding TNALP in either WT or any of the mutations 48 h after transfection. This was either due to rapid secretion or protein degradation (data not shown). To determine if homodimeric mutants containing N271Q, specifically N140Q/N271Q and N230Q/N271Q, lead to intracellular retention, transfected cells were cultured for 16 h with 1 μM of the proteasome inhibitor MG-132. The culture medium was collected, and TNALP-Flag was pulled down with Flag-immunoprecipitation and the cells were lysed and analyzed with immunoblotting. The immunoblot analysis revealed that TNALP-Flag was localized intracellularly in the N140Q/N271Q and N230Q/N271Q (fold-change 1.0 (range 0.8–1.2) and 1.3 (0.9–1.7) *versus* WT, respectively) in comparison with the corresponding extracellular levels (fold-change 0.1 (0–0.1) and 0 (0–0)) (Fig. 4).

**Table 2:** Immunoblot of homodimeric mutants, pooled samples (n = 6) after Flag-affinity purification of TNALP-Flag from cell media

Homodimeric mutation	Molecular weight (kDa)		Fold-change (vs WT)
	TNALP-flag	Flag-peptide	
WT	84	8	1.0
N140Q	83	8	0.4
N230Q	84	8	0.5
N271Q	78	8	0.4
N303Q	80	8	0.5
N430D	81	8	0.5
N140Q/N230Q	72	8	0.5
N140Q/N271Q	72	8	0.2
N140Q/N303Q	73	8	0.4
N140Q/N430D	72	8	0.6
N230Q/N271Q	–	8	0.0
N230Q/N303Q	68	7	0.4
N230Q/N430D	70	7	0.6
N271Q/N303Q	68	7	0.4
N271Q/N430D	66	7	0.7
N303Q/N430D	66	9	0.7

### The N-glycans seem to stabilize the protein loops, including the Ca^2+^-binding domain

**Figure 5:**
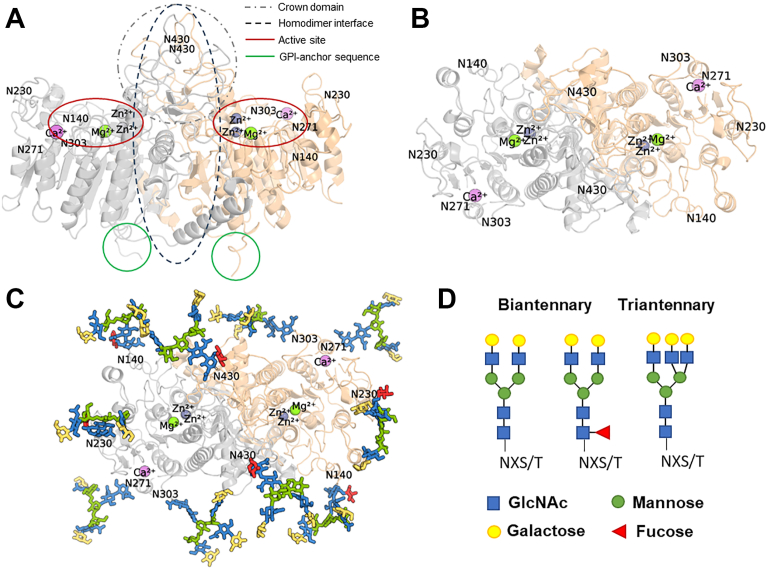
**Protein and N-linked glycan model of the human TNALP homodimer.***A* and *B,* homodimer of the crystal structure of human TNALP (PDB: 7YIV) with one Ca^2+^ (*purple*), 1 Mg^2+^ (*green*) and two Zn^2+^ (*gray*), as viewed from the side (*A*) and top (*B*). *C,* top view of TNALP with designated N-glycan sites modeled using CHARMM-GUI. *D,* structure of bi- and triantennary complex-type N-glycans used for molecular dynamic simulations, colors of glycans between panels *B* and *D* are matched. TNALP, tissue-nonspecific alkaline phosphatase.

**Figure 6:**
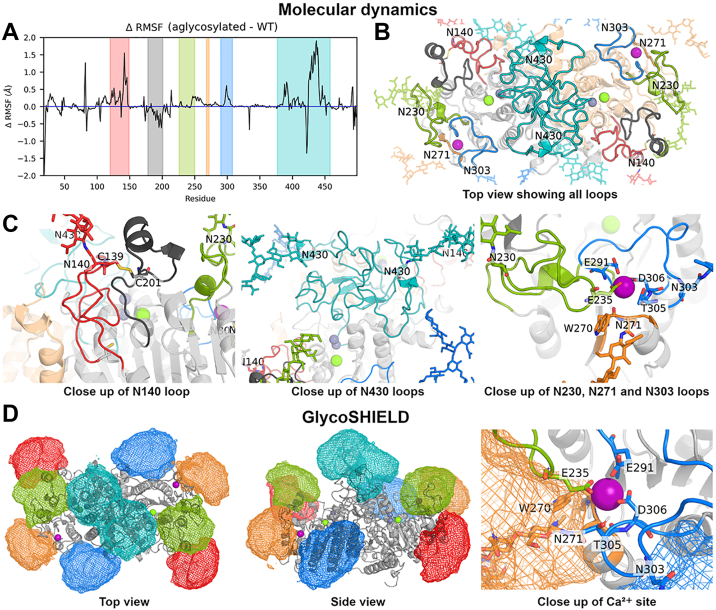
**Molecular dynamics and GlycoSHIELD models of human TNALP.***A,* average per-residue RMSF difference (ΔRMSF) between aglycosylated and fully glycosylated (WT) TNALP from molecular dynamics simulation, colored section reflects protein loop colors in the figure. *B,* top view of the TNALP homodimer, loops and their attached glycan are colored as follows: N140 (*red*), N230 (*green*), N271 (*orange*), N303 (*blue*), and N430 (*teal*) and the protein section between Y178-C201 (*dark gray*). *C*, A close-up view of the loop containing N140 and a disulfide bridge connected Y178-C201 segment (*left*), the loop dimer containing N430 (*middle*), and the loops containing N230, N271 and N303 (*right*). *D*, average density from N-glycan conformations sampled using the GlycoSHIELD tool as viewed from the top (*left*) and side (*middle*), (*E*) a close-up view of the Ca^2+-^binding site (*right*). Density colors are matched to the glycans. Ion coloring as follows: Ca^2+^ (*purple*), Mg^2+^ (*green*) and Zn^2+^ (*gray*). TNALP, tissue-nonspecific alkaline phosphatase; RMSF, root mean square fluctuation.

Figure 5, *A* and *B* illustrate the human TNALP dimer with the different domains and the five N-linked glycosylation sites. To investigate how the N-glycans may stabilize the conformations of the human TNALP, we generated a glycosylated protein model containing the most abundant biantennary and triantennary complex-type N-glycans (Fig. 5, *C* and *D*). This, together with an aglycosylated model (where the N-glycan capable residues are changed to glutamine), were investigated using molecular dynamics simulations lasting for 500 ns in triplicate. We conducted a root mean square fluctuation (RMSF) analysis, which measures the average spatial fluctuations experienced by the α-carbon atom of every residue in the simulation. By subtracting the RMSF of the glycosylated (WT) from the aglycosylated protein configurations, we measure which residues fluctuated more in the absence of glycans (positive values) or less (negative values) (ΔRMSF, Fig. 6*A*). We observed that loop sections of TNALP containing the residues N140, N303 and N430 fluctuate more (by 0.5–2 Å) in the absence of a glycan. A section between the loops containing N230 and N271 (R246–K260) also fluctuated more in the absence of glycans. Unlike the other sections, a section encompassing residues Y178–C201 fluctuated by up to 0.5 Å more in the presence of glycans.

To rationalize these observations, a top view of the protein as well as a close-up view of the glycan-attached loops is provided in Figure 6, *B* and *C*, respectively. The Y178–C201 segment is connected to the N140 containing loop *via* a disulfide bond (C139/C201). This specific coordination geometry may explain why glycans seem to stabilize the N140 containing loop but destabilize the Y178–C201 segment. The glycan may pull on the Y178–C201 segment through this covalent connection but provide stabilizing molecular interactions to the N140–containing loop. The loop containing N430 is a large segment (A377–D458) with a short β-strand section (F383–F385 and I413–Y415). Similar to the N140 containing loop, we suggest that the N430-linked glycans provide stabilizing molecular interactions to this large loop segment (Fig. 6*C*).

The loops containing N230, N271 and N303 together form the Ca^2+^-binding domain. The Ca^2+^ is coordinated by residues from these loops, including W270 through its backbone carbonyl group and E235, E291 and D306, through their carboxylate side chains (Fig. 6*E*). Given the elevated RMSF in the loop containing N303 and between the loops containing N230 and N270 in the absence of a glycan, the glycans may act to stabilize Ca^2+^ binding. We note that while N230 and N303 are located on long flexible peptide loops, N271 is located on a short segment (I269–R272) between a α-helix and a β-strand. Further, we note that N271 is much closer to the Ca^2+^ (≈5 Å from the α-carbon) than N230 (≈20 Å) or N303 (≈10 Å), measured in the initial protein conformation.

To extensively sample the conformational landscape of the glycans and observe how glycans can shield certain regions of the protein, we utilized the GlycoSHIELD tool (21). The conformational space of the glycans is illustrated as an ensemble of simulation frames (Fig. S1*A*) and as density maps (Fig. 6, *A* and *B*). Glycan conformations shield the Ca^2+^-binding domain and active sites, with the N271-linked glycan directly covering the Ca^2+^-binding site (Fig. 6*D*). Fig. S1 is provided to show how the lack of specific glycan “de-shields” parts of the protein. This provides a rationale for why specific combinations involving N230 significantly reduced the activity of TNALP (N140Q/N230Q and N230Q/N271Q), while others did not (N230Q/N303Q and N230Q/N430D) (Fig. 1*B*). N230 is flanked by N140 and N271, thus they could make up for its absence even without N303 (N230Q/N303Q) and N430 (N230Q/N430D).

### Effect of single- and double-site mutations on the thermal stability of TNALP

**Figure 7:**
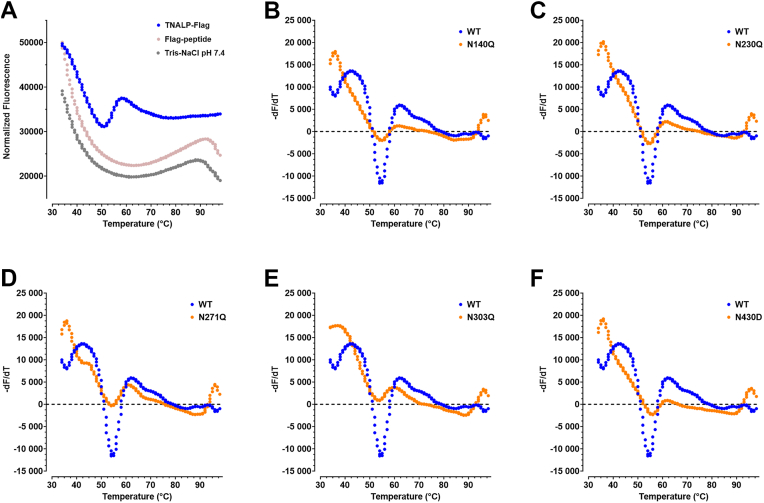
**Thermal shift assay of single-site homodimeric mutations of TNALP.** TNALP-Flag elutions from six replicate transfections were pooled together and the fluorescence intensity of denaturing protein under ramping temperature of 34 °C to 99 °C was measured with SYPRO *Orange*. *A,* normalized fluorescence of Tris-NaCl buffer (pH 7.4), Flag-peptide containing buffer and WT TNALP, showing high initial fluorescence at 34 °C that decreases with increasing temperature. *B* – *F*, first derivative fluorescence of single-site homodimeric mutations *versus* WT TNALP to determine T_m_ of the protein. T_m_, melting temperature; TNALP, tissue-nonspecific alkaline phosphatase.

**Figure 8:**
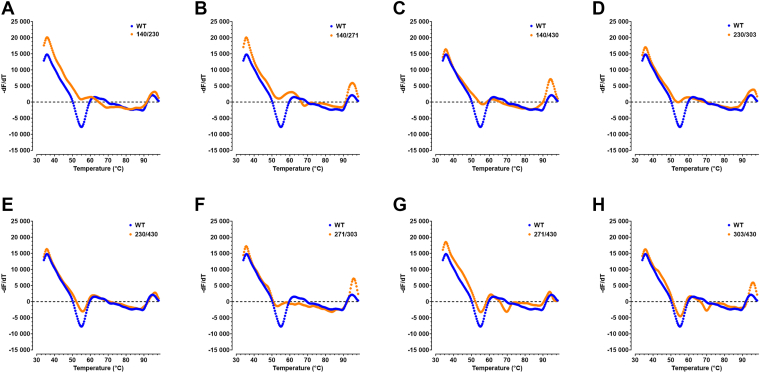
**Thermal shift assay of double-site homodimeric mutations of TNALP.** TNALP-Flag elutions from six replicate transfections were pooled together and the fluorescence intensity of denaturing protein under ramping temperature of 34 °C to 99 °C was measured with SYPRO *Orange*. T_m_ of double-site homodimeric mutations (*orange*) *versus* WT (*blue*) TNALP (*A – H*) is determined from first derivative fluorescence. T_m_, melting temperature; TNALP, tissue-nonspecific alkaline phosphatase.

**Figure 9:**
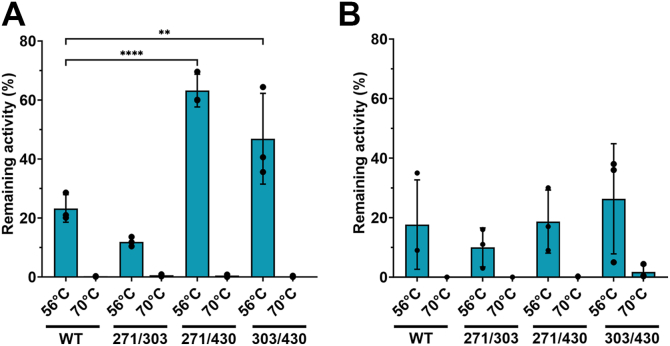
**Heat inactivation of TNALP activity.** (*A*) Homodimeric and (*B*) heterodimeric N271Q/N303Q, N271Q/N430D and N303Q/N430D mutants *versus* WT. Cell supernatants from three independent transfection experiments were subjected for 15 min to 56 °C or 70 °C and the enzymatic activity was measured with p-nitrophenylphosphate assay in diethanolamine buffer at pH 9.8 before and after heat inactivation. The difference in activity before and after heat inactivation is expressed as remaining activity. Mean ± SD, two-way ANOVA with Tukey’s multiple comparisons test. *p* < 0.05 was considered significant. ∗∗*p* = 0.01, ∗∗∗∗*p* < 0.0001. TNALP, tissue-nonspecific alkaline phosphatase.

A thermal shift assay was performed with SYPRO Orange to investigate how glycan site homodimeric mutations influence the thermal stability of the protein. High fluorescence background was observed below 30 °C due to SYPRO Orange dye saturation (Fig. 7*A*). In addition, high initial fluorescence was observed in all samples due to the presence of Flag-peptide in the TNALP sequence. The Flag-peptide has a tryptophan residue, which is possibly exposed on the surface of TNALP and accessible to SYPRO Orange. This led to high fluorescence at the start of the melting curve as observed in the negative control, where free Flag-peptide is present. In order to reduce the interference of the Flag-peptide, the start of the ramping temperature was set at 34 °C. Additionally, first-derivative curve fitting was used to obtain the melting temperature (T_m_), which is defined as the incline in the derivative of fluorescence in relation to the derivative of temperature, where half of the protein molecules reach unfolded state. T_m_ was 54.5 °C for WT, N230Q and N271Q, while for N140Q, N303Q and N430D, T_m_ was 54.9 °C, 53.7 °C and 55.7 °C, respectively (Fig. 7, *B*–*F* and Table 3). Compared to WT, all double-site mutants with N430D showed slightly increased thermal stability (>55 °C), while N271Q/N303Q had decreased thermal stability with T_m_ 52.9 °C. N271Q/N430D and N303Q/N430D showed an additional incline at 69.7 °C and 70.1 °C, respectively, suggesting a two-state protein unfolding (Fig. 8, *E* and *F* and Table 3). To determine possible increased thermal stability of N271Q/N430D and N303Q/N430D, remaining enzymatic activity was measured after 15 min of heat inactivation at 56 °C, 60 °C, 65 °C or 70 °C (Fig. 9). The homodimeric mutants N271Q/N430D and N303Q/N430D showed significantly higher remaining activity in comparison with WT after 56 °C (63 ± 5% and 47 ± 13%, respectively, *versus* 23 ± 4%). This was not the case for N271Q/N303Q (12 ± 1%), which was in agreement with the decreased thermal stability in the thermal shift assay. However, there was no significantly increased thermal stability for the heterodimeric mutants. The enzymatic activity was fully abolished for all samples at 60 °C, 65 °C and 70 °C.

**Table 3:** T_m_ of WT and homodimeric mutants

Homodimeric mutation	T_m_ (°C)
WT	54.5
N140Q	54.9
N230Q	54.5
N271Q	54.5
N303Q	53.7
N430D	55.7
N140Q/N230Q	54.9
N140Q/N271Q	53.7
N140Q/N430D	56.5
N230Q/N303Q	54.1
N230Q/N430D	55.7
N271Q/N303Q	52.9
N271Q/N430D	55.3, 69.7
N303Q/N430D	55.3, 70.1

### Absence of N-glycan sites did not influence the isoform profile of TNALP

TNALP expressed in mouse osteoblasts comprises four isoforms detected with HPLC (22). We hypothesized that the absence of at least one N-glycan site would affect the bone-specific isoform profile of TNALP. All single-site homodimeric mutants showed the presence of four isoforms with similar retention times as WT. Although four isoform peaks were present in all double-site mutations (except N140Q/N230Q, N140Q/N271Q and N230Q/N271Q) low absorbance levels were observed in most of the double-site mutations due to low enzymatic activity. There was a slight shift to later retention times in N140Q/N430D, N230Q/N430D, N271Q/N303Q, N271Q/N430D and N303Q/N430D in comparison with WT (Fig.S2 and Table S1).

## Discussion

N-linked glycosylation is essential for the enzymatic activity of TNALP (16, 23). However, how the individual N-glycosylation sites influence the stability and enzymatic activity of soluble TNALP remains elusive. Inhibition of global protein glycosylation with tunicamycin during protein synthesis as well as removal of terminal sialic acid with Sialidase A and glycosidase treatment has previously shown to reduce the enzymatic activity of TNALP (16, 24). The results in our study demonstrate that N271Q and all double-site mutations, except N230Q/N303Q and N230Q/N430D, resulted in a significant reduction in enzymatic activity. N271Q, N303Q, N140Q/N271Q, N230/N271Q, N271Q/N430D and N303Q/N430D had a significant DNE, as shown by remaining activity less than 50% of WT when expressing a mutation on one monomer. Absence of N-glycans on one monomer might influence structurally the other monomer which needs to be further studied. Moreover, removing glycans at N140 or N230 together with N271 diminished TNALP secretion and resulted in intracellular TNALP retention, leading to enhanced protein degradation, as cells treated with the proteasome inhibitor MG-132 showed presence of TNALP in the N140Q/N271Q and N230Q/N271Q lysates. In contrast, Komaru *et al.* (14) showed that the removal of a single N-glycan site did not influence the dimerization and activity of membrane-bound TNALP. Furthermore, we observed a larger decrease in activity in both single- and double-site mutations *versus*. WT, in comparison with Komaru *et al.* (14). Similar to our study, these authors found that combined removal of the three sites N230, N271 and N303 decreased the expression and stability of TNALP. N-linked glycosylation may play a crucial role in the function of GPI-cleaved TNALP *versus* GPI-anchored TNALP. As TNALP is a membrane-bound protein, the GPI-anchor, as well as the membrane composition, might be important for protein stabilization during TNALP transportation to the membrane. This might explain the differences between the findings in our study and the findings by Komaru *et al.* (14), but the differences in the stability between glycosylated membrane-bound and soluble TNALP remains to be determined.

Another important implication of N-linked glycosylation in TNALP could be related to the dimerization of TNALP and further oligomerization of the dimers. Diminished dimer stability is related to the mutation N430S, which has been reported in severe infantile HPP (14). However, in the same study, N430D did not affect dimer formation, which suggests that the amino acid, rather than the glycan at this position, has a pivotal role in the dimer interaction located at the crown domain of TNALP. We used the N430D, instead of N430Q in order to preserve enzymatic activity for activity-based detection of TNALP isoforms with HPLC. Furthermore, octamerization of soluble TNALP is evident in the crystalized structure obtained by Yu *et al.* (18). It was, therefore, assumed that membrane-bound TNALP is dimeric, while the cleavage from the GPI-anchor results in oligomerization, which improves the stability of TNALP (18). Nosjean *et al.* (16) have shown that treatment of soluble TNALP with N-glycosidase leads to tetramerization. However, it remains unknown if N-linked glycosylation is essential for preserved dimeric state of soluble TNALP and how it affects protein secretion.

Molecular dynamics simulations show that the presence of glycans stabilizes regions of the protein, especially at or near the loops containing the glycans. GlycoSHIELD modeling of glycan conformations shows a degree of overlap between glycan conformations, specifically between N140, N230 and N271. This suggests that the loss of stabilizing interactions from removing specific glycans can be compensated by others. The implication of this is that the removal of adjacent glycan pairs should impact enzymatic activity more than the removal of non-adjacent pairs. This is supported by mutagenesis showing that mutations of the adjacent pairs N140/N230 and N230/N271 diminished activity more than that of the non-adjacent pairs N230/N303 and N230/N430. However, likely this isn’t the only mechanism at play since glycan removal from the non-adjacent pairs N140/N271 still diminished enzymatic activity. Depending on the location of the glycosylation site on the protein, specific glycans may play a greater role in stabilizing TNALP compared to others.

We observed that the Ca^2+^-binding domain is formed entirely by the loops containing the glycosylation sites N230, N271 and N303. These loops contain three acidic residues, E235, E291 and D306 and their importance in coordinating Ca^2+^ is supported by site-directed mutagenesis (E235G, E291K and D306V) which significantly reduced enzymatic activity. The three sites E235, E291 and D306 bind the Ca^2+^, and R223, Y236 and T305 stabilize the conformation of the Ca^2+^-binding pocket (18, 25). Interestingly, homology comparison of human TNALP with other types of ALP, and specifically the crystal structure of human placental ALP, showed that N271 (N249 in placental ALP) is evolutionarily conserved (26, 27). N249 is one of three glycosylation sites abundant on placental ALP; the crystal structure revealed that N249, stabilized by W248, interacts with a Ca^2+^, forming the Ca^2+^-binding domain (27). Among the glycosylation sites, N271 is the closest to the Ca^2+^, nearly within interacting distance (5 Å from the α-carbon). Furthermore, N271 lies on a short loop between a α-helix and a β-strand, unlike other glycosylation sites on longer, flexible loops. While this was not observed in the simulations, this solvent-exposed α-helix and β-strand section may depend on stabilizing interactions from the N271-glycan to remain firmly attached to the Ca^2+^-binding site. Notably, N271 was the only N-glycan site without core-fucosylated glycans, possibly due to steric effects from the bulky W270 residue to the N-glycan core (3.5 Å), the functional significance of this is yet to be explored (8). This study showed that N271 is important for normal TNALP function and that N271 based on its tight position on a short loop and in proximity to W270 might play an important role in stabilizing Ca^2+^-binding. Although no variants of N271 have been reported, other mutations in the NXS/T sequon have been identified. It has been further confirmed that mutations at the Ca^2+^-binding domain lead to moderate or severe HPP, emphasizing the importance of the integrity of this domain in the normal function of TNALP (18). The pathogenic variants R272C and R272L lead to perinatal HPP, while heterozygous and homozygous R272H lead to adult and infantile HPP, respectively. The T273M variant with unknown significance has been found and described either as benign or likely pathogenic. It remains unclear whether the involvement of the amino acid substitution or the absence of glycosylation is relevant for the pathogenicity of this variant.

Thermal inhibition of ALP is an established method to assess isozyme fractions in the circulation. Heat inactivation at 56 °C leads to partial loss of enzymatic activity of TNALP, where the bone isoform is more heat-sensitive than the liver isoform. However, all serum ALP activity is lost at 65 °C (28). Here, we analyzed the thermal denaturation at ramping temperatures from 30 °C to 99 °C where the T_m_ is determined at the highest fluorescence peak of bound SYPRO Orange to the hydrophobic pockets of the protein during thermal denaturation. The T_m_ of WT TNALP-Flag was determined to be 54.5 °C and only N303Q and N430D shifted the T_m_ with 1 °C, suggesting a slightly more stable protein for N430D and slightly less stable for N303Q. However, a second peak was observed in N271Q/N430D and N303Q/N430D at approx. 70 °C suggesting that these mutations either might lead to a more thermally stable protein or a nonfunctional folded state. A subsequent analysis showed that the homodimeric mutants N271Q/N430D and N303Q/N430D had higher remaining activity than WT and N271Q/N303Q after heating at 56 °C, but the same effect was not observed in the heterodimeric mutants. This is in contrast to the molecular dynamics simulation results, showing that the lack of N-glycans can destabilize the protein. It is possible that under normal conditions, the lack of N-glycans at N271/N430 and N303/N430 may present a barrier to forming a stable active protein conformation. Heating may overcome these barriers and help the mutant-containing proteins to achieve an active conformation. Furthermore, no remaining activity was observed after heat inactivation at 60 °C, 65 °C and 70 °C suggesting the presence of protein in a non-functional folded state between 56 °C and 70 °C.

Circulating TNALP in the bloodstream in human beings is composed of three bone and three liver isoforms. An additional bone isoform, normally expressed in osteoblasts, was found in 60% of patients with chronic kidney disease and is a potential biomarker of low bone turnover and improved survival in these patients (29, 30). The isoforms are detected in serum samples by HPLC but the structural and functional significance of these isoforms in the circulation is unknown. We hypothesized that the TNALP isoforms might differ in glycan occupancy at each site. However, neither our previous study, where the total pool of TNALP showed to be fully N-glycosylated (8), nor the current study showed evidence to support this hypothesis. Removal of neither single nor multiple sites altered the isoform profile for the mutations that showed enzymatic activity. More studies are needed to elucidate the role of N-linked glycosylation in the presence of different TNALP isoforms in the circulation.

The strength of this study lies in the combined approach, integrating experimental protein analyses with novel computational simulations of glycosylation-induced structural changes. However, the low protein yield after transfection, which led to pooling samples for immunoblotting and thermal shift assay, is a limitation. The high variation in both enzymatic activity and protein expression may be explained by normalization to protein concentration but not to cell density or viability, although we kept constant both the plasmid concentration and the number of cells for transfection. Although the enzymatic activity assay has high sensitivity, the buffer composition, including divalent ion concentration, might affect the structural conformation of TNALP. For example, we used constant amounts of Zn^2+^ and Mg^2+^ in the activity assay but did not adjust the Ca^2+^ concentration. This, on the other hand, might lead to non-physiological, stabilizing or de-stabilizing intermolecular interactions, influencing the enzymatic activity, which needs to be further investigated. Furthermore, we generated heterodimeric mutations to determine DNE. Expression of a mixture of mutated and WT plasmid should theoretically lead to mutated TNALP heterodimers, however, this has not been practically confirmed. Therefore, the increased remaining activity might be derived from the presence of WT homodimer, rather than structural influence of the mutated monomer, which is a limitation to this study. However, this effect can be explained by loss of stability of one monomer that would lead to loss-of-function rather than structurally affecting the WT monomer or DNE. For example, in the N230Q/N271Q mutants, where no extracellular release and subsequently no activity was observed in the homodimeric mutants, the activity in the mutant heterodimers might be partially rescued by WT homodimerization and extracellular release. Therefore, the enhanced activity of the mutant heterodimers compared to mutant homodimers might be due to favored WT homodimerization. On the other hand, large thermal shifts for the other homodimeric mutants were not observed, which might indicate stable mutant monomers and WT-mutant heterodimerization is still possible with partial DNE. Still, the assumption that the mutations lead to less stable monomers, which would promote a favored WT homodimerization, indicates that the glycosylation sites are important for increased dimer stability, regardless of the ratio of homo- and heterodimers. However, WT-mutant heterodimerization for more accurate DNE needs to be further confirmed. The molecular dynamics simulation enabled sampling local differences in protein stability in the presence or absence of N-glycans. However, they did not enable us to visualize larger-scale conformational changes, *i.e.*, related to protein folding. Given that the conformations of large N-glycans are not expected to converge from a microsecond-scale simulation (31), convergence was not expected from our 500 ns simulations. This is illustrated in Fig. S3, which shows that while the overall conformational spaces were similar, the N-glycan distributions were asymmetric between the two protein subunits. To address this issue and obtain a more complete distribution, we utilized GlycoSHIELD, which exhaustively samples glycan orientations and revealed a comparable symmetric distribution. Although that we investigated protein folding through thermal stability shifts, further investigations are required for structural determination of the effect of glycosylation on TNALP folding.

In conclusion, single and double N-glycan-site mutations led to reduced secretion and enzymatic activity of soluble TNALP. N271 showed a central role in preserving enzymatic activity and protein release in combination with N140 and N230. N-glycans at N271, N230 and N303 are also involved in the formation of the Ca^2+^-binding domain. A dynamic interaction between the N-glycans has a shielding role in providing a functional protein fold of TNALP. The presence of different TNALP isoforms is not dependent on a single N-glycan site and the implication of N-linked glycosylation in the heterogeneity of circulating TNALP isoforms needs to be evaluated further. Additional studies are required to explore possible differences in the enzymatic activity and protein stability between membrane-anchored and soluble TNALP.

## Experimental procedures

### Site-directed mutagenesis and plasmid isolation

A cDNA encoding human TNALP (UniProt: P05186, a.a. 1–489) cloned into a pcDNA3 vector was used to generate substitution mutations of the N-linked glycosylation sites of TNALP. The GPI-anchor gene region was substituted with a Flag-peptide at the C-terminal of TNALP to produce soluble epitope-tagged human TNALP (*set*-hTNALP-pcDNA3) (32). The five N-glycosylation sites located at asparagine (N) 140, 230, 271, 303 and 430 were substituted with glutamine (Q) or aspartic acid (D) to produce the mutations N140Q, N230Q, N271Q, N303Q and N430D. The mutations were generated in single-primer PCR reactions with forward and reverse primers as described by Edelheit *et al.* (33). The PCR reaction containing *set*-hTNALP-pcDNA3 plasmid, Q5 High-Fidelity DNA polymerase (New England BioLabs), dNTP (Thermo Fisher Scientific) and forward or reverse primers (Table 4) was run in T100 Thermal Cycler (Bio-Rad Laboratories). The residual non-mutated parental plasmid was digested with DpnI restriction enzyme (Thermo Fisher Scientific) for 16 h at 37 °C.

**Table 4:** Primer design for site-directed mutagenesis

Primer	Primer sequence
N140Q	5′-GTTCCCGGTGCCAAACCACCCAGG-3′
N230Q	5′-CATGTACCCCAAGCAGAAAACTGATGTGGAGTATGAGAG-3′
N271Q	5′-CACTTCATCTGGCAACGCACGGAACTCC-3′
N303Q	5′-CTGAACAGGAACCAAGTGACGGACCCGTC-3′
N430D	5′-CGGTGAACGAGAGGATGTCTCCATGGTGG-3′
Sequencing forward	5′-ATGTCATCATGTTCCTG-3′
Sequencing reverse	5′-TAGTTCTGCTCGTGGAC-3′

The obtained mutant and wild-type *set*-hTNALP-pcDNA3 were amplified in *Escherichia coli* DH5α by heat-shock at 42 °C for 30 s and cultured on LB agar plate with 100 μg/ml Ampicillin overnight at 37 °C. Colonies were selected, further amplified in selection medium with 100 μg/ml Ampicillin and the plasmids were purified with GenElute HP Plasmid Miniprep Kit (Merck KGaA). Mutated plasmids were verified with Sanger sequencing (Eurofins Analytik GmbH) and mutations were confirmed with Multiple Sequence Alignment Tool by EMBL-EBI (available at https://www.ebi.ac.uk/) by aligning the sequenced strands with the coding sequence of TNALP (*Homo sapiens* ALP, biomineralization associated (*ALPL*), transcript variant 1, mRNA; NCBI Reference Sequence NM_000478.6). The confirmed mutated plasmids were further amplified and purified with GenElute HP Plasmid Midiprep Kit (Merck), diluted in aliquots of 300 to 500 ng/μl in nuclease-free water and stored at −20 °C.

### Expression of soluble TNALP with N-glycan site mutations in mouse calvarial osteoblasts

The isolation and culture of mouse calvarial osteoblasts from *Alpl*^*+/−*^ mice have been described in detail elsewhere (8, 34, 35). The isolation procedure was conducted with approval of the Institutional Animal Care and Use Committees of Sanford Burnham Prebys Medical Discovery Institute. The osteoblasts were SV40 T-antigen immortalized, clone selected and cryopreserved for further experiments (36). Briefly, cells were cultured in Dulbecco's modified Eagles medium (Thermo Fisher Scientific) supplemented with 10% heat-inactivated fetal bovine serum (Merck), 1% GlutaMAX (Thermo Fisher Scientific) and 1% Penicillin/Streptomycin (Merck) in a humidified incubator at 37 °C with 5% CO_2_ and tested for *mycoplasma* contamination with *Mycoplasma* check (Eurofins Analytik GmbH).

TNALP with single-, double and triple-site homodimeric mutations or a WT-to-mutated plasmid in a 1:1 ratio (heterodimeric mutations) were transfected with Nucleofector kit V (Lonza Group) according to the manufacturer's instructions. An empty vector pcDNA3-Flag (EV) was used as a negative control. The transfected cells were cultured in 6-well plates in DMEM with 1% fetal bovine serum at 37 °C with 5% CO_2,_ and the supernatant was harvested after 2 days and centrifuged at 2500*g* for 10 min to remove cellular debris. EDTA-free protease inhibitor cocktail was added to the supernatant, and the samples were stored at −20 °C until further analysis.

For the determination of intracellular retention and proteasome degradation of the expressed TNALP, transfected cells with WT, mutated plasmid or EV control were cultured with 1 μM MG-132 (Merck) for 16 h in 6-well plates. The cells were scraped in PBS, pelleted and lysed with Pierce RIPA buffer (Thermo Fisher Scientific) with brief sonication. The cell lysates were stored at −80 °C for immunoblotting analysis.

### TNALP activity assay

TNALP activity was assessed with p-nitrophenylphosphate enzymatic assays as previously described (37). Briefly, cell supernatant was diluted 1:10 in buffer containing 12.5 mM Tris, 12.5 mM sodium bicarbonate and 10 μM zinc acetate, pH 8.5 and added in 1:5 in the wells of a 96-well plate with 150 mM sodium chloride. TNALP substrate solution containing 1 M diethanolamine, 1 mM magnesium chloride and 10 mM p-nitrophenylphosphate, pH 9.8 was added to the wells and the absorbance was measured at 405 nm directly and every 5 min up to 30 min on Multiscan ELISA plate reader (Thermo Fisher Scientific). The enzymatic activity is calculated as mU/well according to the equation mU/well = ((Abs_30_-Abs_0_)∗1000∗tot well Vol)/(6∗30 min). The enzymatic activity was normalized to the protein concentration measured with the BCA Protein Assay Kit (Thermo Fisher Scientific).

### Capillary-based immunoblotting

TNALP was purified with the Flag immunoprecipitation kit (Merck) according to the instructions. TNALP was eluted with 3× Flag-peptide (Merck) in 50 mM Tris-HCl, 150 mM sodium chloride, pH 7.4. All elutions from the pulled-down extracellular TNALP-Flag from six replicate transfections with homodimeric mutations were pooled together, vacuum-concentrated (except the empty vector control, where elutions of three transfection were used). Protein concentration was measured with the BCA assay (Thermo Fisher Scientific) and 50 μg/ml of sample was loaded in each well for immunoblotting. Cell lysates and extracellular TNALP-Flag in the cell culture medium were prepared in a replicate transfection experiment of single- and double-site homodimeric mutations containing N271Q. The prepared purified extracellular TNALP-Flag and cell lysates were immunoblotted with capillary-based immunoassay on Jess Automated Western Blot System according to the manufacturer’s instructions (ProteinSimple; Bio-Techne). Briefly, the sample was mixed in 1:4 with 5× Fluorescent Mastermix, containing 200 mM dithiothreitol, and heated to 95 °C for 5 min and cooled on ice. Rabbit polyclonal anti-Flag (F7425, Merck, lot 0000120996) or mouse monoclonal anti-Flag M2 (F3165, Merck, lot 0000405712) antibody and mouse anti-actin monoclonal antibody (ACTN05, Invitrogen, lot WI3205973) were used for TNALP-Flag as well as Flag-peptide and actin detection, respectively. Samples, biotinylated ladder, diluted primary antibodies in Antibody Diluent 2 (1:20), supplied anti-rabbit or anti-mouse secondary HRP-conjugated antibody and luminol-peroxide solution (1:1) were loaded in a customized plate and run on a 12 to 230 kDa fluorescence separation module. The results were analyzed with Compass for SW software version 7.0.0 (Bio-Techne). The chemiluminescence intensity was normalized to Flag-peptide in the extracellular TNALP-Flag and to actin in the cell lysates. The mutations were further normalized to the WT control and the expression shown as fold-change of WT. A negative control of empty vector transfected cells was used to verify specific TNALP-Flag expression.

### Molecular dynamics simulation

The TNALP dimer structure was extracted from the most recent cryo-EM structure from the Protein Data Bank (PDB-ID: 7YIV) (18) (residues L18–H496 of the protein were used). The CHARMM-GUI (38) web server was used to assign the protonation states of the protein at pH 8, add glycans and place the protein in a simulation water box. Glycans, found to be most abundant on all sites from a previous study (8), were added to N140, N230, N271, N303 and N430 (WT). An aglycosylated configuration of the protein was also made where these residues where *in silico* mutated to glutamine. Both a fully glycosylated (WT) and an aglycosylated configurations were placed in a 17 × 17 × 17 Å water box containing 150 mM NaCl and excess Na^+^ to neutralize overall charge. The simulation boxes were equilibrated using the default CHARMM-GUI equilibration protocol (38). Following this, production simulations were conducted at 310K for 500 ns in triplicate using the GROMACS program (39). Each replica was initiated with a unique random velocity seed drawn from a Maxwell distribution at 310 K. Default CHARMM-GUI simulation settings were used for the equilibration and production steps (38). Protein RMSF was analyzed using the MDAnalysis program (40). The first 100 ns of each replica were discarded as equilibration. RMSF was calculated based on α-carbon atoms and averaged across protein subunits from each simulation replica. The RMSF values from the glycosylated and aglycosylated protein configurations were subtracted to obtain the ΔRMSF.

### GlycoSHIELD modeling

The GlycoSHIELD tool (21) was used to model a large conformational ensemble of the glycans on N140, N230, N271, N303 and N430 on the TNALP dimer. As above, the most abundant forms of glycans on these residues were used, except for N303 where the “Gal3_Fuc1” glycan, containing a core-fucose was used as an unfucosylated version was not available. As the core-fucose is located at the base of the glycan, this should not affect the conformational space sampled for N303. Conformations were generated using the “GlycoSHIELD.py” script with the coarse-grained mode, the “--shuffle-sugar” option, and the recommended threshold value of 3.5 Å for checking glycan-protein overlap. This script generates conformations for each residue separately. To combine the conformations into a single trajectory file, the “GlycoTRAJ.py” script was run with the “--maxframe" option set to 1000. The VolMap tool implemented in VMD (41) was used to generate average densities for the glycan conformation; the densities were illustrated using the PyMOL program (The PyMOL Molecular Graphics System, Version 2.5 Schrödinger LLC).

### Thermal shift assay

Purified wild-type TNALP and TNALP with single- and double-site homodimeric mutations were freeze-dried, re-suspended in deionized water and analyzed with protein thermal shift assay. Protein Thermal Shift Dye Kit (Life Technologies) was used according to the manufacturer's instructions. One microgram of protein was mixed in 1:10 with 500 mM Tris, 1.5 M NaCl, pH 7.4. The dissolved protein was then mixed on ice with protein thermal shift buffer and 8× SYPRO Orange dye in a 1:5 ratio. Four replicate samples were pipetted in a 96-well PCR plate and the plate was covered with optically clear sealing film, briefly spun down and placed on ice. The fluorescence intensity was read in the StepOne Plus PCR instrument (Life Technologies) with ramp temperature increase from 34 °C to 99 °C. T_m_ was obtained from the normalized fluorescence intensity by first-derivative curve fitting in GraphPad Prism version 10.0.2 (GraphPad Software Inc., La Jolla). Protein buffer containing Flag-peptide was used as a negative control.

### Heat inactivation

To determine the effect of thermal denaturation on the enzymatic activity of TNALP, cell supernatants from homodimeric and heterodimeric N271Q/N303Q, N271Q/N430D and N303Q/N430D mutants or WT were diluted 1:10 in 150 mM sodium chloride. The samples were divided in five equal parts – one part set aside for activity prior to heat inactivation and four parts for incubation at 56 °C, 60 °C, 65 °C and 70 °C for 15 min. The enzymatic activity was measured using the TNALP activity assay as described above, and the remaining activity was determined by subtracting the activity after heat inactivation from that before heat inactivation.

### TNALP isoform profiling

The isoform profile of the expressed TNALP was determined by HPLC as reported elsewhere (29). Purified soluble TNALP with N-glycan-site homodimeric mutations or WT was diluted 1:100 in 150 mM sodium chloride to obtain activity <200 U/L. The diluted sample was injected and the isoforms were separated in a weak anion-exchange column (SynChropak AX300, Eprogen Inc.) under a gradient of 0.6 M sodium acetate at 30 °C. Detection was performed by online mixing with 1.8 mM p-nitrophenylphosphate in buffer with 250 mM diethanolamine, 6 g/L Triton X-405 and 1 mM magnesium chloride, pH 10.1, at 37 °C in a packed-bed postcolumn, with p-nitrophenol product detection at 405 nm. The isoforms were illustrated as individual peaks and the area under each peak was integrated and presented as percentage of the total area.

## Statistical analysis

Expression of homodimeric and heterodimeric mutations was performed in six biological replicates. Mean, standard deviation and confidence intervals were calculated for TNALP activity and remaining activity was expressed as activity of the mutations subtracted by the activity of WT. One-way ANOVA with Dunnett’s multiple comparisons test for TNALP activity and two-way ANOVA with Tukey’s multiple comparisons test for remaining activity after 56 °C and 70 °C heat inactivation was performed with *p*-value <0.05 set as significant.

## Data availability

The data that support the findings of this study are available from the corresponding author upon reasonable request.

## Supporting information

This article contains supporting information (Figs. S1, S2 and S3 and Table S1).

## Declaration of generative AI and AI-assisted technologies in the writing process

During the preparation of this article, the authors used Microsoft Copilot in order to condense and improve sentence structure in the abstract and discussion. After using this tool/service, the authors reviewed and edited the content as needed and take full responsibility for the content of the published article.

## Conflict of interest

The authors declare that they have no conflicts of interest with the contents of this article.
