# Supplementary material for: N-linked glycosylation plays an essential role in the stability and function of tissue-nonspecific alkaline phosphatase
Source: J Biol Chem. 2025 Dec 20;302(2):111092. doi: 10.1016/j.jbc.2025.111092 (PMC12861225; doi:10.1016/j.jbc.2025.111092)
Supplement: Supporting information [file mmc1.pdf]

Supporting Information

N-linked glycosylation plays an essential role in the stability and function of tissue-nonspecific alkaline phosphatase

Diana Atanasova, Ali Saad Kusay, Lavanya Moparthi, Stefan Koch, Mathias Haarhaus, Sonoko Narisawa, José Luis Millán, Eva Landberg, Per Magnusson

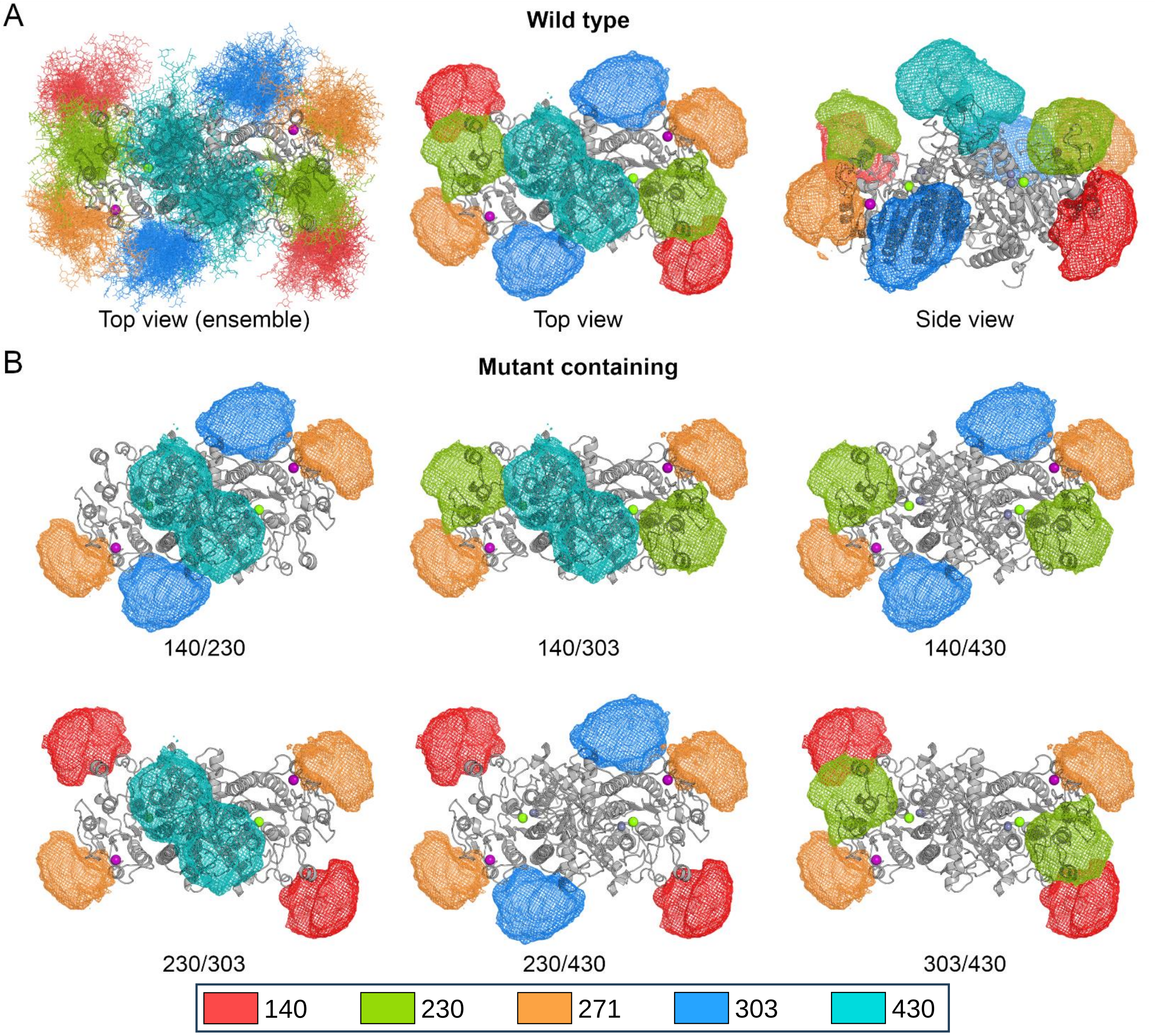

Figure S1. Illustration of glycan conformations generated by the GlycoSHIELD tool. (A) Ensemble of 50 N-glycan conformations selected uniformly from 1000 total conformations for each N-glycan (*left*), average density of from all glycan conformations as viewed from the top (*middle*) and from the side (*right*). (B) TNALP proteins containing double mutants (not involving N271), the conformational density for each mutant pair is hidden for illustration. Coloring follows: 140 (red), 230 (green), 271 (orange), 303 (blue) and 430 (teal). Ion coloring as follows: Ca^{2+} (purple), Mg^{2+} (green) and Zn^{2+} (grey).

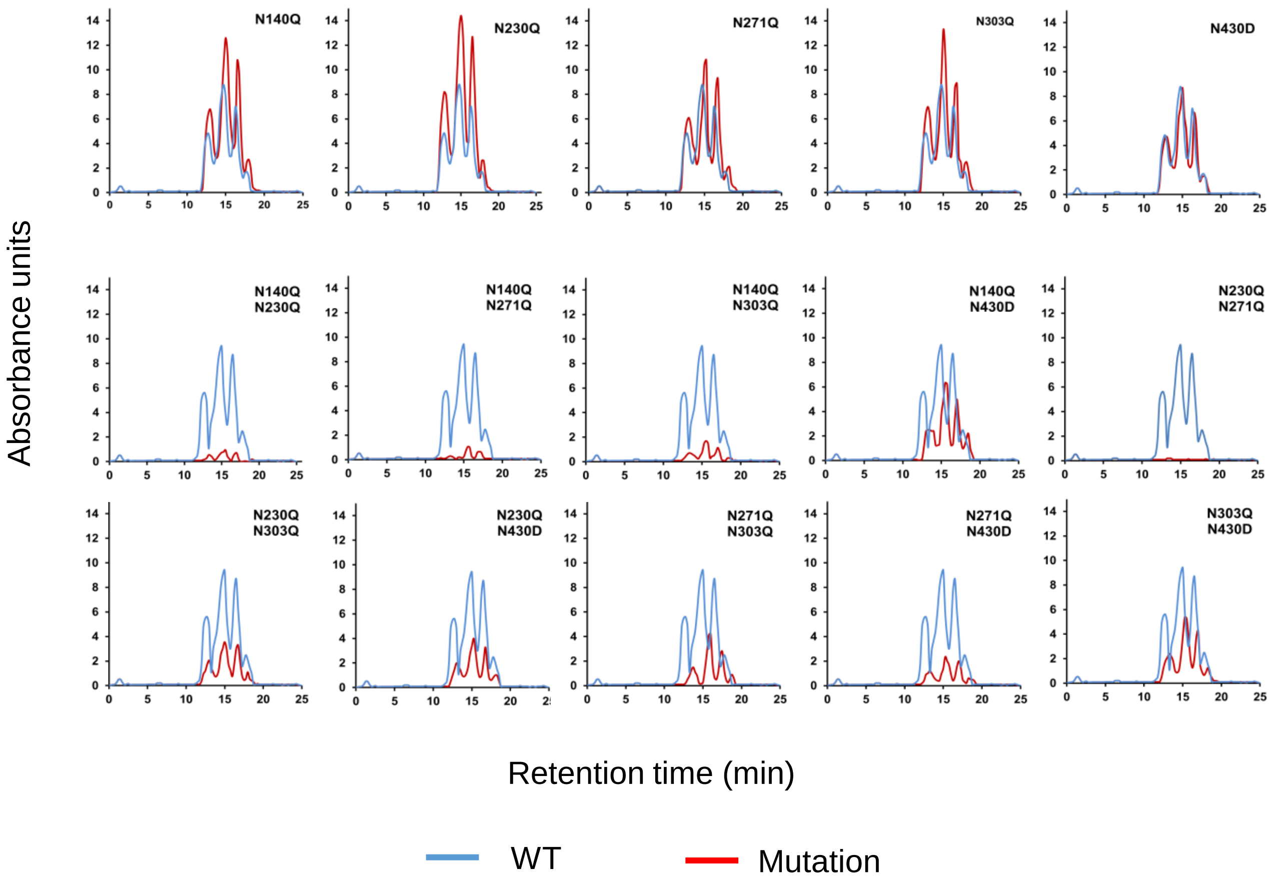

Figure S2. TNALP isoform profiles of dimeric N-glycan site mutations. Cellular supernatants were collected 48 hours after transfection with wild-type (WT) or dimeric single- or double-site mutations. The TNALP isoforms were separated with high performance liquid chromatography (HPLC) with weak anion exchange column and post-column reaction with p-nitrophenylphosphate. The absorbance of the product p-nitrophenol is measured at 405 nm and each peak corresponds to one TNALP isoform with a specific retention time.

Table S1. TNALP isoform profiles from HPLC

	Isoform 1 (%)	Isoform 2 (%)	Isoform 3 (%)	Isoform 4 (%)	Total TNALP activity (U/L)
WT	25	45	25	5	182
N140Q	24	42	27	7	143
N230Q	25	43	26	6	127
N271Q	26	41	27	6	102
N303Q	25	43	25	7	135
N430D	26	43	25	6	144
N140Q/N230Q	30	43	23	4	19
N140Q/N271Q	34	44	22	0	14
N140Q/N303Q	29	44	24	3	37
N140Q/N430D	24	41	27	8	122
N230Q/N271Q	0	0	0	0	0
N230Q/N303Q	23	41	28	8	63
N230Q/N430D	24	42	26	8	64
N271Q/N303Q	26	40	27	7	72
N271Q/N430D	25	40	27	8	50
N303Q/N430D	23	42	27	8	100

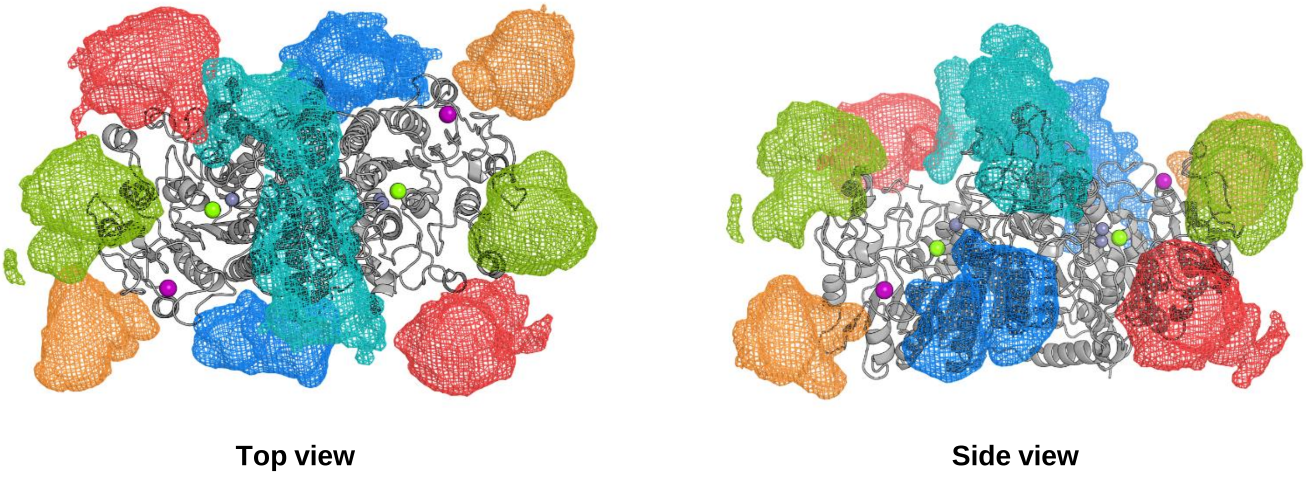

Figure S3. Illustration of N-glycan conformational space from molecular dynamics simulations. Average density from N-glycan conformations sampled from 100-500 ns of triplicate molecular dynamics simulations, as viewed from the top (*left*) and side (*right*). Density colors are matched to the glycans: N140 (red), N230 (green), N271 (orange), N303 (blue) and N430 (teal).
